# Predictive value of systemic immune-inflammation index in the high-grade subtypes components of small-sized lung adenocarcinoma

**DOI:** 10.1186/s13019-024-02528-x

**Published:** 2024-02-01

**Authors:** BoHua Wei, Yi Zhang, Kejian Shi, Xin Jin, Kun Qian, Peilong Zhang, Teng Zhao

**Affiliations:** 1 Department of Thoracic Surgery, Xuanwu Hospital, Capital Medical University, No. 45 Changchun Street, Beijing, China; 2 Laboratory of Respiratory Disease and Thoracic Surgery, KU Leuven, 3000 Leuven, Belgium

**Keywords:** Lung adenocarcinoma, Solid, Micropapillary, Systemic immune-inflammation index, Nomogram

## Abstract

**Background:**

Identification of micropapillary and solid subtypes components in small-sized (≤ 2 cm) lung adenocarcinoma plays a crucial role in determining optimal surgical procedures. This study aims to propose a straightforward prediction method utilizing preoperative available indicators.

**Methods:**

From January 2019 to July 2022, 341 consecutive patients with small-sized lung adenocarcinoma who underwent curative resection in thoracic surgery department of Xuanwu Hospital, Capital Medical University were retrospectively analyzed. The patients were divided into two groups based on whether solid or micropapillary components ≥ 5% or not (S/MP5+ and S/MP5-). Univariate analysis and multivariate logistic regression analysis were utilized to identify independent predictors of S/MP5+. Then a nomogram was constructed to intuitively show the results. Finally, the calibration curve with a 1000 bootstrap resampling and the receiver operating characteristic (ROC) curve were depicted to evaluate its performance.

**Results:**

According to postoperative pathological results, 79 (23.2%) patients were confirmed as S/MP5+ while 262 (76.8%) patients were S/MP5-. Based on multivariate analysis, maximum diameter (*p* = 0.010), consolidation tumor ratio (CTR) (*p* < 0.001) and systemic immune-inflammation index (SII) (*p* < 0.001) were identified as three independent risk factors and incorporated into the nomogram. The calibration curve showed good concordance between the predicted and actual probability of S/MP5+. Besides, the model showed certain discrimination, with an area under ROC curve of 0.893.

**Conclusions:**

The model constructed based on SII is a practical tool to predict high-grade subtypes components of small-sized lung adenocarcinoma preoperatively and contribute to determine the optimal surgical approach.

## Introduction

Lung cancer is the leading cause of cancer-related death and the second most commonly diagnosed malignancy worldwide [1]. Currently, adenocarcinoma (ADC) is the most common histologic type. In 2011, International Association for the Study of Lung Cancer/American Thoracic Society/European Respiratory Society (IASLC/ATS/ERS) further classified lung adenocarcinoma into five pathological subtypes: lepidic, acinar, papillary, micropapillary and solid patterns [2]. Micropapillary and solid components were considered to be high risk and closely associated with poorer prognosis [3, 4].

Lobectomy with mediastinal lymphadenectomy has been recommended as the standard surgical treatment of early-stage non-small cell lung cancer (NSCLC) for a long time. While recent studies have confirmed segmentectomy could be another option for tumors of 2 cm or smaller [5, 6]. However, Nitadori and colleagues proved that for tumors with MIP component ≥ 5%, patients treated with lobectomy had a reduced risk of recurrence compared with those treated with limited resection [7]. A similar conclusion had been reached by Su and colleagues. For lung ADCs presenting as pure solid nodules, segmentectomy was significantly associated with worse recurrence-free survival (RFS) and overall survival (OS) in patients with MIP component > 5% than lobectomy [8]. Besides, both studies confirmed that for tumors with MIP < 5%, the prognosis of patients underwent lobectomy or segmentectomy was similar. In addition, as a relatively new metastasis mode, spread through air spaces (STAS) had been found to be associated with the presence of minor solid or micropapillary components [9], and lobectomy had been proven to be associated with better outcomes in early stage STAS-positive lung adenocarcinoma [10]. Above all, we suppose that a threshold of 5% for high-risk subtype components may be crucial for the selection of surgical procedures for small-sized lung adenocarcinoma. That is to say lobectomy should be more considered in a selected ADC patients group with lesion diameter ≤ 2 cm but MIP or solid components ≥ 5%. On the other hand, segmentectomy may be the optimal surgical procedure for patients whose high-grade subtypes components are less than 5%. Therefore, it’s of great value to predict high-grade subtypes components of small-sized adenocarcinoma preoperatively. However, few studies have focused on it.

Systemic immune-inflammation index (SII), which was first developed in 2014 is an easily accessible inflammatory parameter which can reflect the general immune status of the body [11]. Despite being greatly affected by infections, autoimmune diseases and many other factors, it has still been confirmed of great value for early diagnosis, staging, predicting curative effect and prognosis of lung cancer [12–14]. Nevertheless, few studies have focused on its potential role in predicting pathological subtypes components. Nomogram has been considered as an intuitive method to describe the generating probability of an event since its first application [15]. To sum up, we intend to construct a nomogram to predict whether high-grade subtypes components are more than 5% or not in lung adenocarcinoma ≤ 2 cm to help select the optimal surgical procedure preoperatively based on SII and other conventional indicators.

## Materials and methods

This study is reported in accordance with the Strengthening the Reporting of Observational Studies in Epidemiology (STROBE) statement [16].

### Ethical statement

Our study was approved by Institutional Review Board of Xuanwu Hospital, Capital Medical University (KS2022141, approved on July 27, 2022). Written informed content was obtained from all enrolled patients.

### Patients

From January 2019 to July 2022, a total of 427 patients underwent lobectomy or segmentectomy due to lung nodule ≤ 2 cm in our institution. Finally, data of 341 pathologically confirmed primary invasive lung ADC patients were retrospectively analyzed.

The inclusion criteria for the present study were as follows: (1) Preoperative thin-section chest CT revealed a solitary nodule ≤ 2 cm clinically considered as stage IA lung cancer and pathologically confirmed as primary invasive adenocarcinoma; (2) had a clear description of histological patterns; (3) received lobectomy or segmentectomy with systemic lymph node dissection or sampling; (4) had complete blood cell parameters within 5 days before operation; We excluded patients: (1) clinically with obvious N1 and N2, or distant metastasis; (2) postoperative pathological results suggested presence of invasive adenocarcinoma variants (including mucinous, enteric, or fetal morphologies, etc.); (3) Pathology confirmed as adenocarcinoma in situ (AIS) or minimally invasive adenocarcinoma (MIA); (4) had clinical evidence of acute infection or other inflammatory conditions within 2 weeks before admission; (5) received preoperative chemotherapy, radiotherapy, targeted therapy or other treatment; (6) had a history of other malignancies; (7) had a history of hematological or immune system disorders; (8) had incomplete clinicopathological data.

### Chest CT scan

Chest CT scan was examined with a window level of -700 Hounsfield Unit (HU) and a window width of 1500 HU as the lung window. The mediastinal window was defined as a window level of 40 HU and window width of 350 HU. The maximum diameter of the nodule was measured on the lung window. Consolidation tumor ratio (CTR) was calculated as the maximum diameter of the lesion on the mediastinal window divided by the maximum diameter on the lung window.

### Peripheral blood cell parameters

All blood samples were drawn in a fasting state within 5 days before the operation and stored in collection tubes containing ethylene diamine tetraacetic acid (EDTA). The complete blood count test was analyzed by Sysmex XE 5000 automated hematology analyzer. SII was calculated as the counts of platelets × neutrophils/lymphocytes in peripheral blood.

### Measurement of carcinoembryonic antigen (CEA)

Blood samples were collected in fasting state too, stored in polyester gel vacuum blood collection tubes and sent to the laboratory department within 2 h. Then centrifuged at 3000r/min with a radius of 10 cm. After 10 min of centrifugation, serum was taken and detected using Roche carcinoembryonic antigen detection kit (Roche Diagnostics GmbH) according to the principle of double antibody sandwich method. The normal range is below 5 ng/ml.

### Histological subtype evaluation

For histological evaluation, 10% formalin-fixed and paraffin-embedded tumor tissues were cut and stained with hematoxylin and eosin. Then the subtypes of the tumor were categorized based on the 2011 IASLC/ATS/ERS classification by two pathologists. All subtypes of resected tumors were categorized as lepidic, acinar, papillary, solid, or micropapillary. The component of each subtype was recorded in 5% increments. The lesions were cut every 3 mm to create paraffin blocks and all pathology reports were accomplished after reviewing slides as thoroughly as possible. For the analysis, we grouped tumors with 5% or more solid or micropapillary (S/MP) components as S/MP5+. Tumors with less than 5% S/MP components were grouped as S/MP5−.

### Statistical analysis

IBM SPSS (version 26.0) and R software (version 4.0.3) were used for statistical analysis. Continuous variables were provided as medians (interquartile ranges [IQRs]) if the distribution was nonnormal, and as means ± standard deviation (SDs) if the distribution was normal. Mann–Whitney u-test or student t-test was used for comparing the differences between the two groups for continuous variables. Categorical variables were presented as numbers and percentages and were analyzed with chi-square or Fisher’s exact test. Univariate and multivariate logistic regression analyses were performed to explore the potential factors related to S/MP5+. Odds ratio (OR) with a 95% confidence interval (CI) was used to estimate correlation strength. Subsequently, a nomogram was constructed based on multivariate analysis. Its performance was evaluated by the calibration plot and the receiver operating characteristic (ROC) curve. Besides, it was subjected to a 1000 bootstrap resampling validation to evaluate its performance. Specifically, randomly and repeatedly select the same size of patients as the entire cohort from the original data set to construct a bootstrap sample. The model derived from the original cohort is fit to the new bootstrap sample. Then repeating this process 1000 times and it would produce 1000 model performance indices. The average value of the indices is considered the bias-corrected estimate of the model’s performance. A two-sided *p*-value < 0.05 was considered statistically significant among all statistical analyses.

## Results

**Fig. 1:**
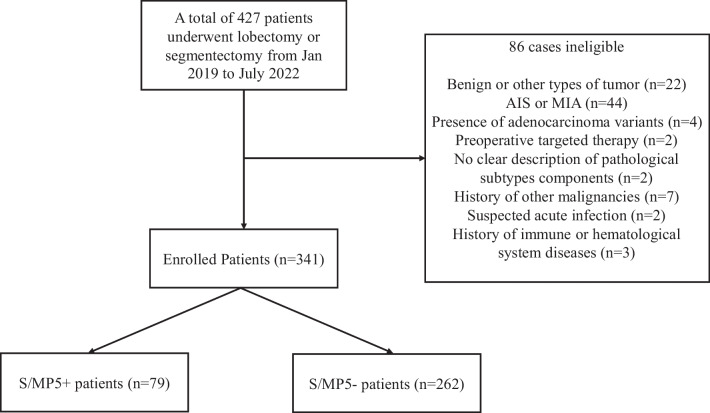
The summary of screening enrolled patients

**Fig. 2:**
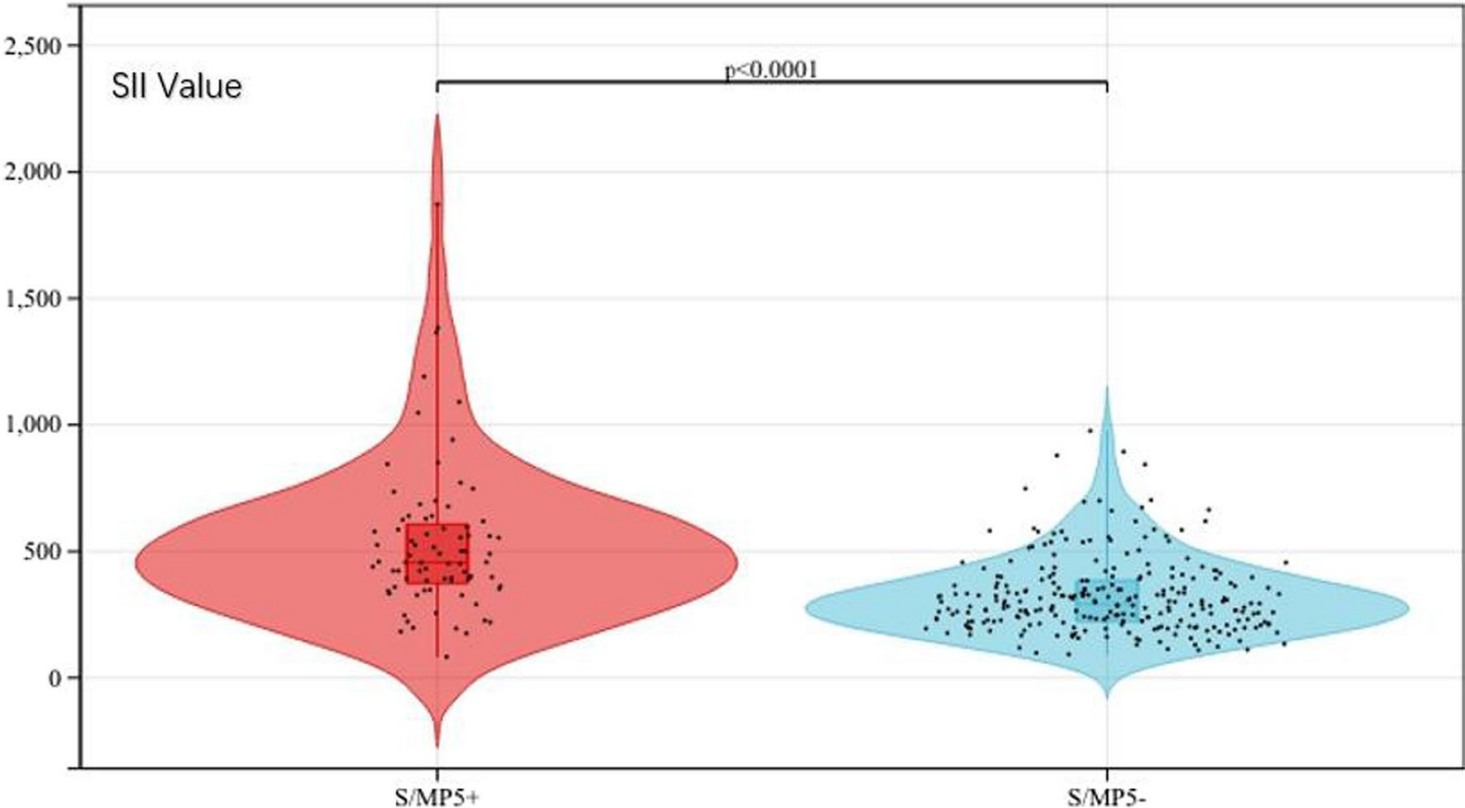
The violin plot of SII values in patients with different high-grade subtypes components

The specific process of enrolling patients is shown in Fig. 1. Of all enrolled patients, a total of 79 (23.2%) patients were confirmed as S/MP5+ and 262 (76.8%) patients as S/MP5−. Compared with S/MP5− patients, S/MP5+ patients had larger tumor size (1.70 cm vs. 1.51 cm, *p* < 0.001), higher CTR (*p* < 0.001) and higher frequency of abnormal CEA (19.0% vs. 9.5%, *p* = 0.022). Moreover, SII was significantly higher in S/MP5+ group than S/MP5- group (531.70 ± 292.27 vs. 323.44 ± 147.92, *p* < 0.001) (Fig. 2). As for pathological features, S/MP5+ patients had higher Ki-67 index (*p* < 0.001), higher frequency of pleural invasion (48.1% vs. 26.6%, *p* < 0.001), lymphovascular invasion (13.9% vs. 0.4%, *p* < 0.001), STAS (29.1% vs. 1.5%, *p* < 0.001) and lymph node metastasis (20.3% vs. 1.5%, *p* < 0.001). Thus, S/MP5 + patients presented with a more advanced pathology stage (*p* < 0.001). Additionally, comparing the surgical procedures in the two groups we found that compared with S/MP5- group, more patients in S/MP5+ group underwent lobectomy (88.6% vs. 67.2%, *p* < 0.001). There was no significant difference in age, gender and smoking history between S/MP5+ and S/MP5- patients. The specific comparison of preoperative factors and surgical characteristics in different high-grade subtypes patients were respectively shown in Table 1 and 2.

**Table 1:** The comparison of preoperative factors in patients with different high-grade subtypes components

Variables	Total (n = 341)	S/MP5+ (n = 79)	S/MP5− (n = 262)	*p*-value
Age	59.27 ± 9.64	59.28 ± 8.76	59.26 ± 9.90	0.990
Gender				0.118
Male	134 (39.3%)	37 (46.8%)	97 (37.0%)	
Female	207 (60.7%)	42 (53.2%)	165 (63.0%)	
Smoking history				0.200
Yes	89 (26.1%)	25 (31.6%)	64 (24.4%)	
No	252 (73.9%)	54 (68.4%)	198 (75.6%)	
Diameter	1.55 ± 0.32	1.70 ± 0.25	1.51 ± 0.32	< 0.001*
CTR	0.16 [0, 0.52]	0.71 [0.40, 0.87]	0.09 [0, 0.29]	< 0.001*
CEA				0.022*
> 5	40 (11.7%)	15 (19.0%)	25 (9.5%)	
≤ 5	301 (88.3%)	64 (81.0%)	237 (90.5%)	
SII	371.68 ± 210.09	531.70 ± 292.27	323.44 ± 147.92	< 0.001*

*CTR* Consolidation tumor ratio, *CEA* Carcinoembryonic antigen, *SII* Systemic immune-inflammation index,* *p*-value<0.05

**Table 2:** The comparison of surgical characteristics in patients with different high-grade subtypes components

Variables	Total (n = 341)	S/MP5 + (n = 79)	S/MP5− (n = 262)	*p*-value
Surgical procedures				< 0.001*
Lobectomy	246 (72.1%)	70 (88.6%)	176 (67.2%)	
Segmentectomy	95 (27.9%)	9 (11.4%)	86 (32.8%)	
Ki-67 value (%)	10 [5–20]	20 [10, 40]	10 [5–10]	< 0.001*
Pleural invasion				< 0.001*
Yes	93 (27.3%)	38 (48.1%)	55 (26.6%)	
No	248 (72.7%)	41 (51.9%)	207 (73.4%)	
Lymphovascular invasion				< 0.001*
Yes	12 (3.5%)	11 (13.9%)	1 (0.4%)	
No	329 (96.5%)	68 (86.1%)	261 (99.6%)	
STAS				< 0.001*
Yes	27 (7.9%)	23 (29.1%)	4 (1.5%)	
No	314 (92.1%)	56 (70.9%)	258 (98.5%)	
Lymph node metastasis				< 0.001*
Yes	20 (5.9%)	16 (20.3%)	4 (1.5%)	
No	321 (94.1%)	63 (79.7%)	258 (98.5%)	
Pathology stage				< 0.001*
IA	240 (70.4%)	34 (43.1%)	206 (78.6%)	
IB	81 (23.8%)	29 (36.7%)	52 (19.8%)	
IIB	9 (2.6%)	8 (10.1%)	1 (0.4%)	
IIIA	11 (3.2%)	8 (10.1%)	3 (1.2%)	

*STAS* Spread through air spaces, * *p*-value<0.05

Subsequently, the four objective and preoperatively accessible indicators (Diameter, CTR, CEA and SII) were incorporated into multivariate logistic regression analysis. We found that diameter (OR 5.176, 95%CI 1.476–18.154, *p* = 0.010), CTR (OR 45.827, 95%CI 15.222–137.960, *p* < 0.001) and SII (OR 1.005, 95%CI 1.003–1.007, *p* < 0.001) were three independent predictors of S/MP5 + (Table 3).

**Table 3:** Multivariate logistic regression analysis of predictors for S/MP5+

Variables	OR	95%CI	*p*-value
Diameter	5.176	1.476, 18.154	0.010*
CEA			
≤ 5	1 (Ref)	–	–
> 5	0.567	0.196, 1.643	0.731
CTR	45.827	15.222, 137.960	< 0.001*
SII	1.005	1.003, 1.007	< 0.001*

*OR* Odds ratio, *CI* Confidence interval, * *p*-value<0.05

**Fig. 3:**
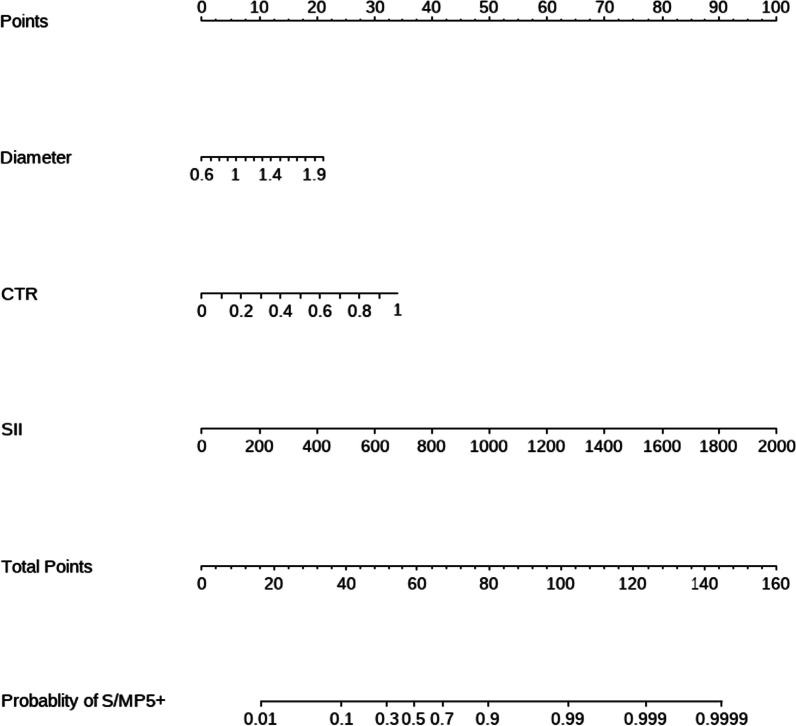
The nomogram to predict the probability of S/MP5 + in small-sized lung adenocarcinoma

**Fig. 4:**
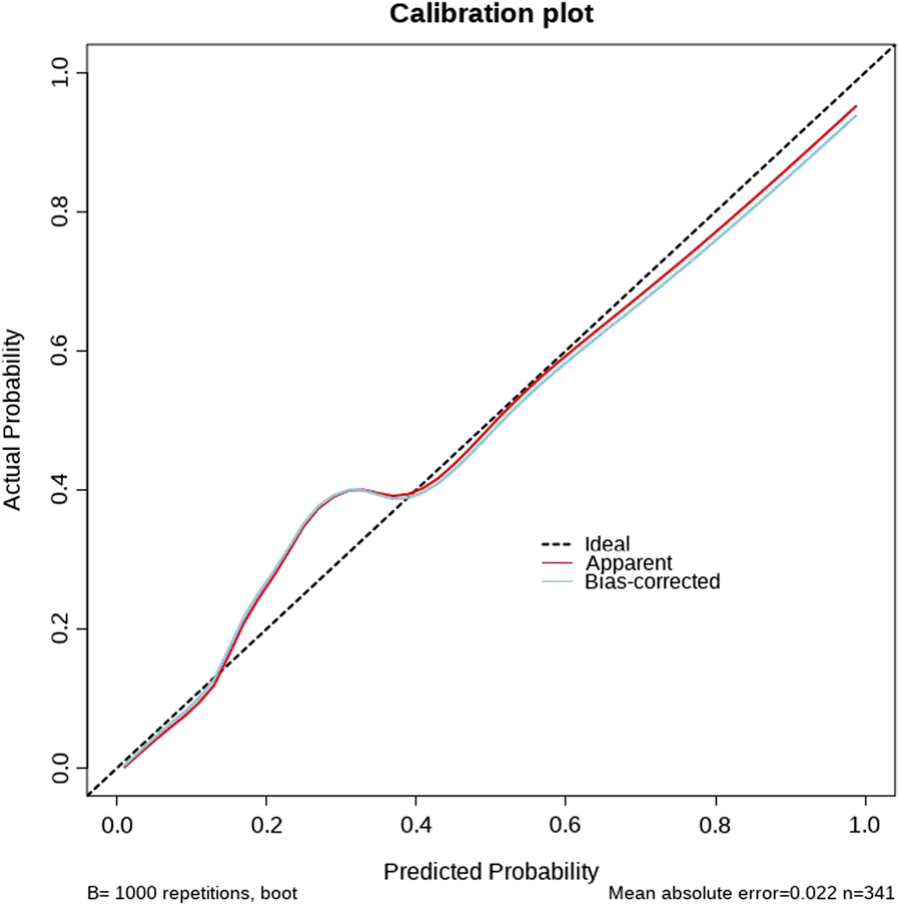
The calibration plot of the nomogram

**Fig. 5:**
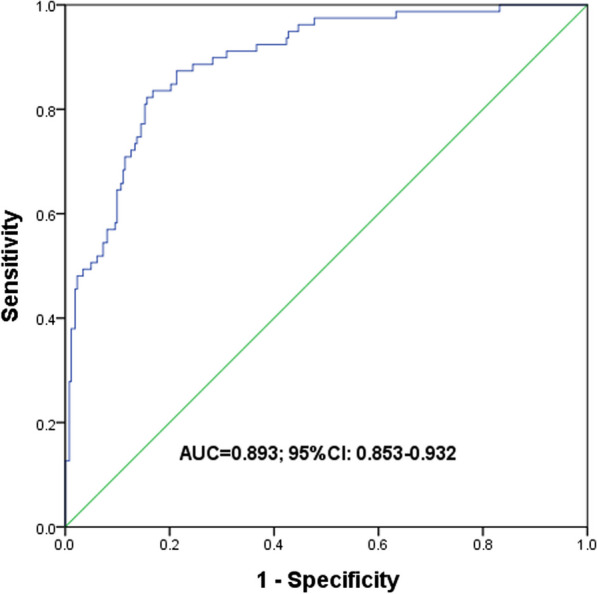
The ROC curve of the model

In order to intuitively show the results and guide clinical practice, we constructed a nomogram based on multivariate analysis (Fig. 3). Besides, the nomogram was subjected to a 1000 bootstrap resampling for internal validation and a minor mean error (0.022) was achieved. Then a calibration plot was depicted to comparing the predicted and actual possibility of S/MP5+. Perfect prediction would correspond to the 45° dashed line and relatively good concordance was obtained in both apparent and bias-corrected curve from the entire cohort and bootstrapping respectively (Fig. 4). Finally, we evaluated the discrimination of the model by ROC curve and certain discrimination was found with an area under curve (AUC) of 0.893 (95%CI 0.853–0.932) (Fig. 5). It means the model can discern a patient with an event from a patient without an event 89.3% of the time.

For every patient, the value of each variable was given a score on the point scale axis. The total score could be calculated by adding every single score together. Locate it on the total points axis and then draw a line straight down to get the exact probability of S/MP5+.

The X-axis and Y-axis respectively represented the predicted and actual probability of S/MP5+. A perfect prediction model would correspond to the black dotted line. The red and blue full line respectively represented the apparent derived from the entire cohort and bias-corrected curve by bootstrapping.

## Discussion

In recent years, segmentectomy is gradually accepted as an alternative surgical procedure for lobectomy of small-sized NSCLC. However, previous studies have confirmed for adenocarcinoma ≤ 2 cm with 5% or more high-grades subtypes components, segmentectomy is associated with higher frequency of recurrence compared with lobectomy. Thus, lobectomy still should be more considered for this part of patients. Therefore, it’s of great value to predict whether high-grade subtypes components higher than 5% in small-sized adenocarcinoma preoperatively. In this retrospective study, we built a nomogram to try to solve this problem based on CT diameter, CTR and SII. The nomogram demonstrated relatively good performance through calibration plot and ROC curve. For patients with a solitary nodule smaller than 2 cm suspected of lung adenocarcinoma, the model may be useful for selecting the optimal surgical procedure from the perspective of pathological subtypes components. However, the limitations of our model should also be addressed. Nomogram itself has the shortcoming of overfitting. Thus, the discrimination can vary greatly if applied to different cohorts, despite excellent discrimination was observed in our derivation cohort. Therefore, the generalizability of our model still need external validation using data from other centers.

Compared with S/MP5- patients, S/MP5+ patients had a higher frequency of pleural invasion, lymphovascular invasion, lymph node metastasis and STAS, which was consistent with the results -of previous studies [9, 17, 18]. Therefore, relatively poorer prognosis of S/MP5+ patients was foreseeable due to differences in these high-risk factors even though long-term follow-up has not been completed yet.

Most of the patients (72.1%) enrolled in the present study underwent lobectomy. However, further analysis of patients with different high-grade subtypes components revealed that a significantly higher proportion of S/MP5+ underwent lobectomy compared with S/MP5- patients. We supposed it was mainly related to the larger tumor sizes and the higher CTR. Lobectomy might be more likely selected considering the higher rate of invasive adenocarcinoma and poorer prognosis [19, 20]. However, from the perspective of postoperative pathology, our selection of surgical approaches remained conservative. 176 (67.2%) S/MP5− patients underwent lobectomy while segmentectomy might be the optimal choice for these patients. On the other hand, 9 patients underwent segmentectomy but confirmed as S/MP5+. Lobectomy might be more beneficial for improving the prognosis of these patients.

The most important predictor in our model was SII. Nevertheless, it had some inevitable limitations due to its role as an inflammatory index. It is greatly affected by infections, drugs, other malignancies, hematological disease or even exercise and weather [21, 22]. Thus, combining with other possible indicators may further improve predictive accuracy and practicality. Frozen section (FS) is another commonly used means to identify pathological subtypes during the operation. However, its sensitivity of identifying minor high-grade subtypes components was low according to previous studies [23, 24]. Moreover, obtaining tissue for FS is difficult in many cases limited by the location of the lesion. Imaging is another promising approach. We have found CTR which based on CT an independent predictor of S/MP5+ in the present study. ^18^F-fluorodeoxyglucose positron emission tomography/computed tomography (FDG-PET/CT) is a commonly used tool in the management of cancer patients. A study conducted by Chang and colleagues revealed that for lung adenocarcinoma ≤ 3 cm, maximum standard uptake value (SUVmax), metabolic tumor volume (MTV) and total lesion glycolysis (TLG) were all significantly higher in patients with minor high-grade subtypes components than negative patients [25]. However, due to the low incidence of lymph node or distant metastasis and the expensive fees, PET/CT is rarely performed in patients with tumors less than 2 cm in our institution. Therefore, other simple imaging features to predict subtypes components still requires further investigation.

In 2020, a new grading system for invasive pulmonary adenocarcinoma was proposed by the International Association for the Study of Lung Cancer Pathology Committee [26]. In this new grading system, lung adenocarcinomas were classified into 3 grades based on the predominant and high-grade histologic pattern with a cutoff of 20%. However, we didn’t apply it as our theoretical basis mainly because of its different inclusion criteria. Stage I cases were enrolled in that study. Thus, some tumors between 2 and 4 cm might be included while cases with lymph node metastasis were excluded. Besides, the study did not provide surgical approaches for patients with different grades. Therefore, we still based our research on high-grade patterns with a cutoff of 5%.

Inflammation serves an important role in tumor progression, invasion and metastasis [27]. Neutrophils promote tumor progression by releasing angiogenic factors, including vascular endothelial growth factor (VEGF), matrix metalloproteinase 9 (MMP-9) and prokineticin-2 (PROK2) [28]. Platelets have been proven to protect tumor cells from shear forces and assault of NK cells and stimulate tumor cell proliferation to form micro metastasis foci. Meanwhile, platelet-derived growth factors help to open the capillary endothelium to accelerate tumor cell extravasation [29]. Thus, neutrophils and platelets are considered primarily pro-tumor. On the contrary, lymphocytes are mainly responsible for combating external infection, clearing variant cells in the body, and exerting inhibitory effects on tumor generation and progression. Hence, either an increase in the number of platelets or neutrophils or a decrease in the number of lymphocytes leads to an elevation of SII, indicating higher invasiveness of the tumor, consistent with the general cognition of each pathological subtype of lung adenocarcinoma. However, the specific mechanisms of why solid or micropapillary components cause such changes are not very clear and require further exploration.

In addition to the shortcomings of nomogram itself mentioned before, several limitations should also be pointed out in our study. First of all, the retrospective nature and limited samples of our study therefore may introduce some inevitable selection bias. Besides, we just analyzed total lymphocyte count to calculate SII rather than lymphocyte subsets. It has been proved that CD3 + T lymphocytes, CD4 + T lymphocytes and the rate of CD4/CD8 are closely correlated with the prognosis of lung cancer [30]. We suspect more satisfactory results may be obtained by applying the data of each subpopulation. In addition, we didn’t perform prognostic follow-up for the included patients due to our small sample size, especially the small number of patients who underwent segmentectomy in the S/MP5+ group. Above all, multicenter researches with larger sample size are needed to further optimize our model and improve its practicality in the future. On the other hand, the specific mechanism by which the increase in SII and the presence of high-grade subtypes components is still unclear. Further researches on lymphocyte subsets or analysis of tumor microenvironment in tumor specimens may be helpful.

## Conclusions

High-grade subtypes components are important in determining the surgical approach for small-sized adenocarcinoma. SII, CTR and diameter was three simple predictors of the presence of 5% or more micropapillary or solid patterns. The nomogram constructed based on them had good efficacy to predict it preoperatively.

## Data Availability

The datasets used and/or analyzed during the current study are available from the corresponding author on reasonable request.

## Abbreviations

ADC: Adenocarcinoma
STAS: Spread through air spaces
SII: Systemic immune-inflammation index
CTR: Consolidation tumor ratio
OR: Odds ratio
CI: Confidence interval
ROC: Receiver operating characteristic
AUC: Area under curve
CEA: Carcinoembryonic antigen
